# School-based physical education, physical activity and sports provision: A concept mapping framework for evaluation

**DOI:** 10.1371/journal.pone.0287505

**Published:** 2023-06-23

**Authors:** Padraic Rocliffe, Brendan T. O’ Keeffe, Ian Sherwin, Patricia Mannix-McNamara, Ciaran MacDonncha

**Affiliations:** 1 Department of Physical Education and Sports Sciences and Health Research Institute, University of Limerick, Limerick, Ireland; 2 School of Education, University of Limerick, Limerick, Ireland; University of Montenegro: Univerzitet Crne Gore, MONTENEGRO

## Abstract

**Objective:**

Physical education, physical activity and sports provision are important factors in whole school health promotion, however, a standardised evaluation framework to evaluate the contribution of these components is lacking. A framework that accounts for the distinct structures and associated factors, that impact upon provision would facilitate a more coherent evaluation.

**Methods:**

A concept mapping methodology, involving the generation of factors relevant to school physical education, physical activity and sports provision and their subsequent thematic and numeric rating and sorting was utilised. Concept mapping effectively gathers, integrates, and visually and numerically represents the composite thinking of a group of relevant and expert stakeholders around a complex social phenomenon. Following a review of the extant literature and synthesis among 20 expert stakeholders, a list of 95 factor statements relevant to school physical education, physical activity and sports provision were developed.

**Results:**

Each factor statement was rated and sorted by 197 multi-disciplinary participants. An eight-cluster framework that demonstrated good validity (stress value: 0.266), was derived from the data based on: 1. Partnerships and Pupil Centered Physical Education; 2. Physical Activity and Sports; 3. School Demographics; 4. Equipment, Facilities and Budget; 5. Extra Costs; 6. Curriculum and Policy; 7. School Management and 8. Timetable. Statements within the cluster on school management received the highest mean importance and modifiability ratings while statements within the cluster on school demographics received the lowest mean importance and modifiability ratings.

**Conclusions:**

Eight overarching structures which account for school physical education, physical activity and sports provision have been identified. Within each of these, structures and overall factors of greatest importance and modifiability have been illuminated. Findings stemming from this rigorous methodology, provide the foundation for the development of a national provision evaluation index to inform both school-level and national policy and actions. It is recommended the current methodology is replicated in other nations to gain corresponding insights.

## Introduction

The physical activity guidelines for adolescents recommend at least an average of sixty-minutes of moderate to vigorous physical activity across the week to optimize a range of health indicators such as body mass index (BMI), cardiovascular fitness and bone health [1, 2]. Despite this, four out of five adolescents globally are physically inactive [3]. Physical activity habits track from adolescences into adulthood underpinning the importance of identifying strategies to promote adolescent physical activity for public health in this phase of life [4]. In addition, physical inactivity, which is now considered a leading contributor to the onset of non-communicable diseases [3], is estimated to cost approximately $300 billion globally by 2030 [5]. Schools have been identified as robust, cost-effective institutions for education and engagement in physical activity to promote health, while simultaneously reducing the international health bill for physical inactivity [6]. Although schools are broadly acknowledged as the most powerful location within which to systematically influence the health of children and adolescents [7], there is a paucity of empirical evidence to support this. Adolescents spend half of their waking day in school and just 20% engage in physical activity outside of school, indicating its unique position to potentiate positive impact [8].

Physical education allows students to “acquire the skills, knowledge and dispositions necessary to be “wise consumers” of physical activity” and sports [9 p3]. Evidence indicates that physical education plays a role in impacting adolescent physical activity behaviour and thus meeting the physical activity guidelines, with one study citing that “each additional MVPA minute in physical education was associated with a 1.4-minute increase of daily MVPA” [10 p604, 11]. The Centre for Disease Control and Prevention (USA) and the Association for Physical Education (UK) have advised that schools target moderate to vigorous physical activity for 50% of physical education class time to optimise adolescent health [12]. Physical education participation is now a requirement in over 90% of schools in Europe, USA and Australia [13, 14] and has subsequently received significant financial investment underlining its importance to adolescent physical activity and health [15, 16]. In response to the World Health Organisation’s (WHO) targets of reducing physical inactivity by 10% in 2025 and 15% in 2030, the International Society for Physical Activity and Health (ISPAH) have identified a whole of school approach to physical activity as one of eight investments that work to promote physical activity, including school physical education, physical activity and sports [17]. Therefore, although physical education is considered the primary vehicle for education and engagement in physical activity, other school physical activity opportunities and sports must also be considered when identifying factors that impact provision. In the context of this study, “provision” refers to the underpinning structures and activities involved in providing the physical education curriculum and opportunities for physical activity and sports participation for adolescents in secondary school education. The extent and nature of the provision reflects the response to the national curriculum, resource base and ethos of a school. To date, there is very little empirical evidence that indicates the factors that impact provision. Despite the significant contribution of school physical activity and sports to obtaining the adolescent physical activity guidelines for health and investment in policy development to advocate for school physical education, physical activity and sports paralleled with the substantial injection of financial resources, a gap in the literature exists that succinctly identifies the factors that impact physical education, physical activity and sports provision in secondary schools.

Using a group concept mapping approach, the aim of this study was to develop a framework of distinct structures and associated factors, which constitutes and impacts on school physical education, physical activity and sports provision. The importance and modifiability of individual factors was also established. The framework will then be utilized to inform the development of the first validated provision evaluation index enabled to measure variation in national and local levels of physical education, physical activity and sports provision in secondary schools. Such an index is essential to establish whether school physical education, physical activity and sports provision: 1) impacts key outcome measures such as physical activity behaviour, health and wellbeing of adolescents, 2) warrants sustained and/or greater financial investment, 3) requires modification of existing provision to potentiate positive impact and 4) provides an impetus for country comparison and benchmarking on key components of provision.

## Materials and methods

### Study design

Group concept mapping has been identified as an effective mixed methodology to underpin the development of a valid, reliable measures and scales by gathering, integrating and visually and numerically representing the composite thinking of a group of relevant stakeholders [18]. Group concept mapping is a standardized procedure that initially requires expert stakeholders to engage in brainstorming exercises, idea generation and idea synthesis [19] to identify a comprehensive list of relevant factors underpinned by the topic of interest (e.g., factors which impact school physical education, physical activity and sports provision). Subsequent tasks require a larger group of participants to rate and sort these factors into clusters that are meaningful to them. A software platform, developed by Concept Systems Inc, was used to facilitate the rating and sorting of the factor statements. These respondents are then visually represented on a two-dimensional concept map via multi-dimensional scaling and hierarchal cluster analysis [19]. Clusters of factors that are located further apart have greater independence than clusters that are closer together [20]. A key strength associated with this methodology is the inclusion of a diverse cohort of participants with multi-disciplinary backgrounds that provide an experiential foundation to inform the group concept mapping analysis [21]. Group concept mapping effectively gathers, integrates and visually and numerically represents the composite thinking of a group of relevant and expert stakeholders around a complex social phenomenon. The group concept mapping process of brainstorming, clustering, rating and mapping relevant factors, is essential to integrate multi-disciplinary stakeholder knowledge and experience into the conceptualization of a complex social phenomenon i.e., distinct structures and factors associated with school physical education, physical activity and sports provision [22].

**Fig 1:**
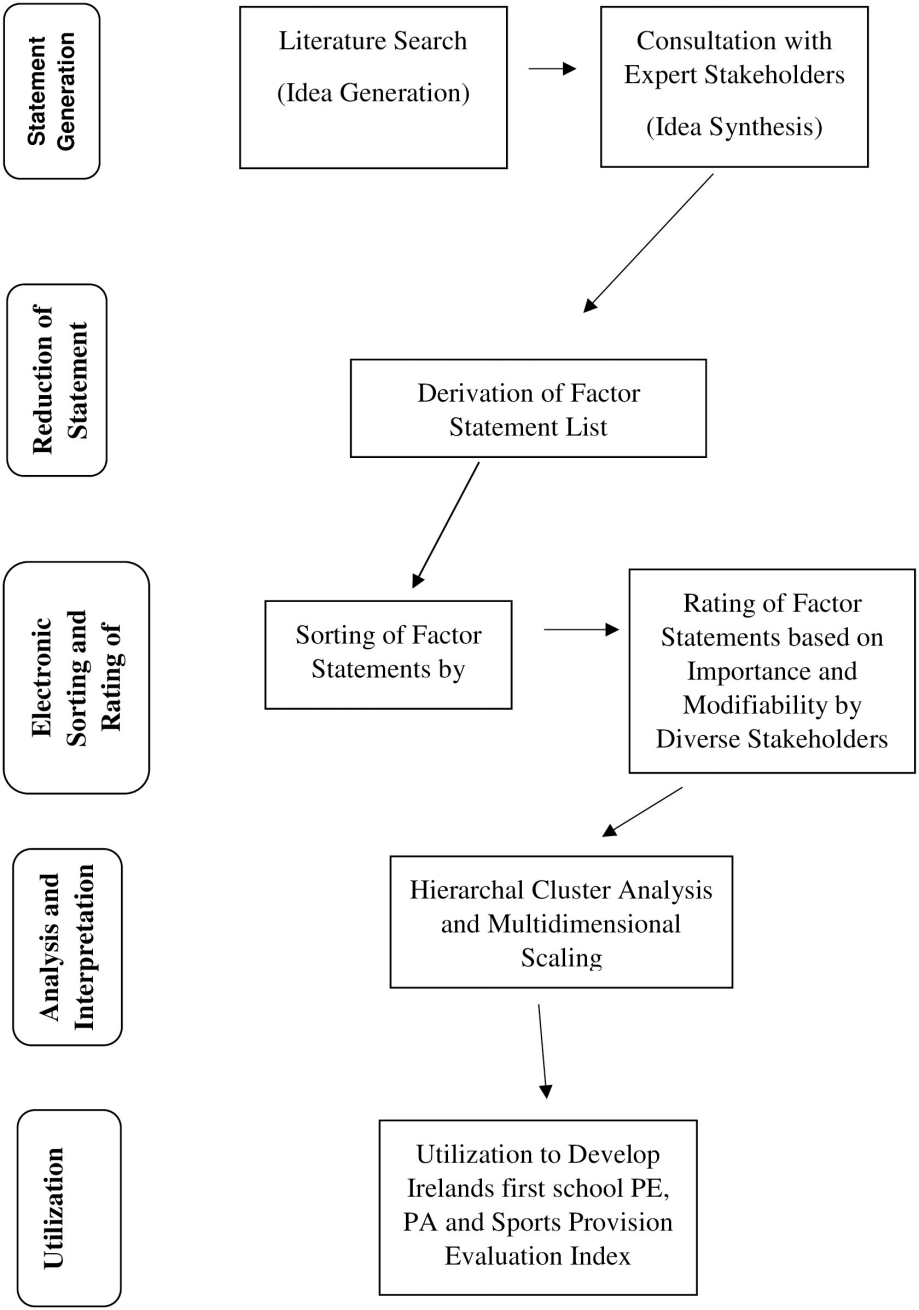
Flowchart of the successive milestones in the group concept mapping methodology.

These successive milestones are illustrated in Fig 1 and are further described in the following sections. Research ethics approval for this study and the associated protocols was granted by the research ethics committee of the Faculty of Education and Health Sciences, University of Limerick, Ireland, V94 XT66.

### Participants

A purposeful sample consisting of 197 respondents were recruited between January 2021 to November 2021 and consented to participate in the sorting and rating of factor statements (20% response rate to invitations). A total of 735 secondary schools in the Republic of Ireland were contacted to engage head physical education teachers, school principals and school sports staff in addition to three leading universities and their associated undergraduate and postgraduate physical education students. National and international experts in the field were also represented to form the overall multi-disciplinary participant sample and expert stakeholder group. All respondents for the participant sample remained anonymous, however, demographic details obtained included the role of participant, gender and nationality, in which the principal investigator had access to and are summarised in Table 1. Responses from the physical education teacher participant sample were representative of school type (boys; girls; mixed) and school size (small; medium; large) in the Republic of Ireland. Upon taking into account study attrition (n = 65) and rejection of data (n = 18) during quality assessment due to improper labelling of clusters, a total of 114 participants contributed to the sorting data. This value far exceeds the number of sorters averaged (24.62 ± 15.29) in a group concept mapping review of 69 studies [18]. Participants included physical education teachers (n = 73), undergraduate and postgraduate physical education students (n = 22), national and international experts (n = 17), policy makers (n = 1) and school principals (n = 1). Upon taking into account study attrition (n = 51) and rejection of data (n = 1) due to little variation in the ratings, a total of 145 participants contributed to the rating data on importance (approximately 1.5 times higher than the average participant sample attained) [18]. Participants included physical education teachers (n = 95), undergraduate and postgraduate PE students (n = 27), national and international experts (n = 18), policy makers (n = 2), school principals (n = 2) and school sports support staff (n = 1). Upon taking into account study attrition (n = 63) and rejection of data (n = 1) due to lack of variation in the ratings, a total of 133 participants contributed to the rating data on modifiability (Approximately 2.1 times higher than the average) [18]. Participants included physical education teachers (n = 85), undergraduate and postgraduate physical education students (n = 25), National and International experts (n = 18), policy makers (n = 2), school principals (n = 2) and school sports support staff (n = 1). For the purpose of clarity, Table 2 defines the three aspects of school physical education, physical activity and sports provision outcomes associated with this study.

**Table 1:** Characteristics of participants.

	Sorting (n = 114)	Importance Ratings (n = 145)	Modifiability Ratings (n = 133)
**Gender**	Male (51%), Female (59%)	Male (50%), Female (50%)	Male (50%), Female (50%)
**Current Role**	PE Teacher (64%)	PE Teacher (66%)	PE Teacher (64%)
	Undergraduate/Postgraduate PE Student (19%)	Undergraduate/Postgraduate PE Student (18%)	Undergraduate/Postgraduate PE Student (19%)
	National/International Expert (15%)	National/International Expert (13%)	National/International Expert (14%)
	School Principal (1%)	School Principal (1%)	School Principal (1%)
	Policy Maker (1%)	Policy Maker (1%)	Policy Maker (1%)
		School Sports Support Staff (1%)	School Sports Support Staff (1%)
**Nationality**	Irish (84%)	Irish (85%)	Irish (83%)
	Outside of Ireland (16%) (Italy, Switzerland, Greece, Belgium, Hong Kong, Portugal, Albania, Netherlands)	Outside of Ireland (15%) (Italy, Switzerland, Greece, Belgium, Hong Kong, Portugal, Albania, Netherlands)	Outside of Ireland (17%) (Italy, Switzerland, Greece, Belgium, Hong Kong, Portugal, Albania, Netherlands)

**Table 2:** Description of the provision outcomes.

Provision Outcomes	Description
Physical Education	Includes teaching students a structured curriculum to help them acquire the skills, knowledge, and dispositions necessary to be “wise consumers” of physical activity [9 p3].
Physical Activity	Is any other bodily movement produced by skeletal muscle that result in energy expenditure related to the school setting **(Not Competitions)**, including active recess, active transport, active classroom breaks and extra-curricular physical activities etc.
Sports	Involves participating in or preparing for school sports competitions.

### Factor statement generation

Two methods were utilised to develop an exhaustive list of potential factors that impact school physical education, physical activity and sports provision: a) idea generation and b) idea synthesis. Firstly, idea generation was based upon an extensive literature search for physical education, physical activity and sports provision surveys that were peer reviewed and published in English and formed the basis of external representational validity [23–26,]. Subsequent to this, a prior systematic literature review conducted to explore the current literature on the impact of typical school provision of physical education, physical activity and sports on adolescent physical activity behaviours was utilized [11]. This information was used to underpin the development of a preliminary list of factor statements (n = 110) based on the following focus prompt: “factors that impact physical education, physical activity and sports provision in secondary schools”. Second, idea synthesis took place via the expert stakeholders (n = 20) to qualitatively check for clarity, repetition or need for fragmentation of the preliminary list of factor statements that may impact provision, derived from the literature. The expert stakeholders were selected based on their knowledge and expertise of the subject area. A 5-point Likert scale to evaluate each factor statement (1 = unclear, 5 = clear) was utilized. The expert stakeholders were given the opportunity to record comments on each factor statement related to relevancy, representativeness, rateability, and saturation of the topic area. Aligning with thresholds underpinned by O’Keeffe, factor statements with an evaluation score of below 3 were amended to enhance clarity or were completely removed [27]. Finally, the factor statements were reduced, refined and approved by two authors (PR, CMD) and edits to the list were resolved via consensus. Upon completing idea generation and idea synthesis, a final list of factor statements (n = 95) was randomized and uploaded to Group Wisdom Inc for sorting and rating.

### Sorting and rating

Upon providing participant information and gaining consent, participants were presented with the final list of factor statements in random sequence via the Concept Systems online platform and were asked to sort them into clusters that were meaningful to them. Each factor could only be included in one cluster and participants were encouraged to sort the factors into a minimum of three clusters. There was no maximum limit to how many cluster the participants could include. Participants were also required to rate each factor statement using a 5-point Likert scale on importance (1 = not important, 5 = very important) and modifiability (1 = not modifiable, 5 = very modifiable). Three individual data collection points were conducted, three months apart and the timeframe for completion was set at five weeks for each individual participant. To minimise non-response, bias, a reminder email was circulated to participants at weeks two, four and five.

### Analysis

This study was underpinned by a mixed methods approach whereby qualitative data obtained from the literature/expert stakeholders and quantitative data from the participant sorting and rating were analysed to facilitate the development of the provision evaluation framework. Group Wisdom Inc software was used to conduct multivariate statistical analysis to synthesise the data. Descriptive statistics (means and standard deviations) were calculated for the statement importance and modifiability ratings. The sorting data was computed with multi-dimensional scaling analysis to identify each points location on a two-dimensional concept map. Factor statements that were closer together were sorted together more frequently while statements that were further apart were sorted together less frequently. Internal representational validity was illustrated via an assigned stress value which indicated “the fit of the configuration with the original similarity matrix” [28, p3]. Next, the statements were mapped into clusters using hierarchal cluster analysis. Each cluster indicated conceptual relationships between the statements. The selection criteria included a lower to higher stepwise inclusion strategy from cluster 3 through to 16, illustrated by Kane and Trochim [29]. The expert stakeholders evaluated each cluster and determined the final cluster number in accordance with meaning to ensure there was a distinct set of domains related to factors that may impact school physical education, physical activity and sports provision. Qualitative measures included logically reassigning factor statements that conceptually fit adjacent clusters more appropriately (n = 2). Descriptive statistics for the rating scales on importance and modifiability were reported and used to create Go-Zones (quadrant 1,2,3,4) in which each data point was illustrated on a scatter plot to visualise and demonstrate conceptual relationships among factor statements. Significant differences between the cluster means were established based on a) importance and b) modifiability via a two tailed T-test. The alpha level was set at p<0.05. A detailed description of the methodological approach to the analysis can be found in Kane and Trochim [29].

## Results

### Sorting/cluster map

**Fig 2:**
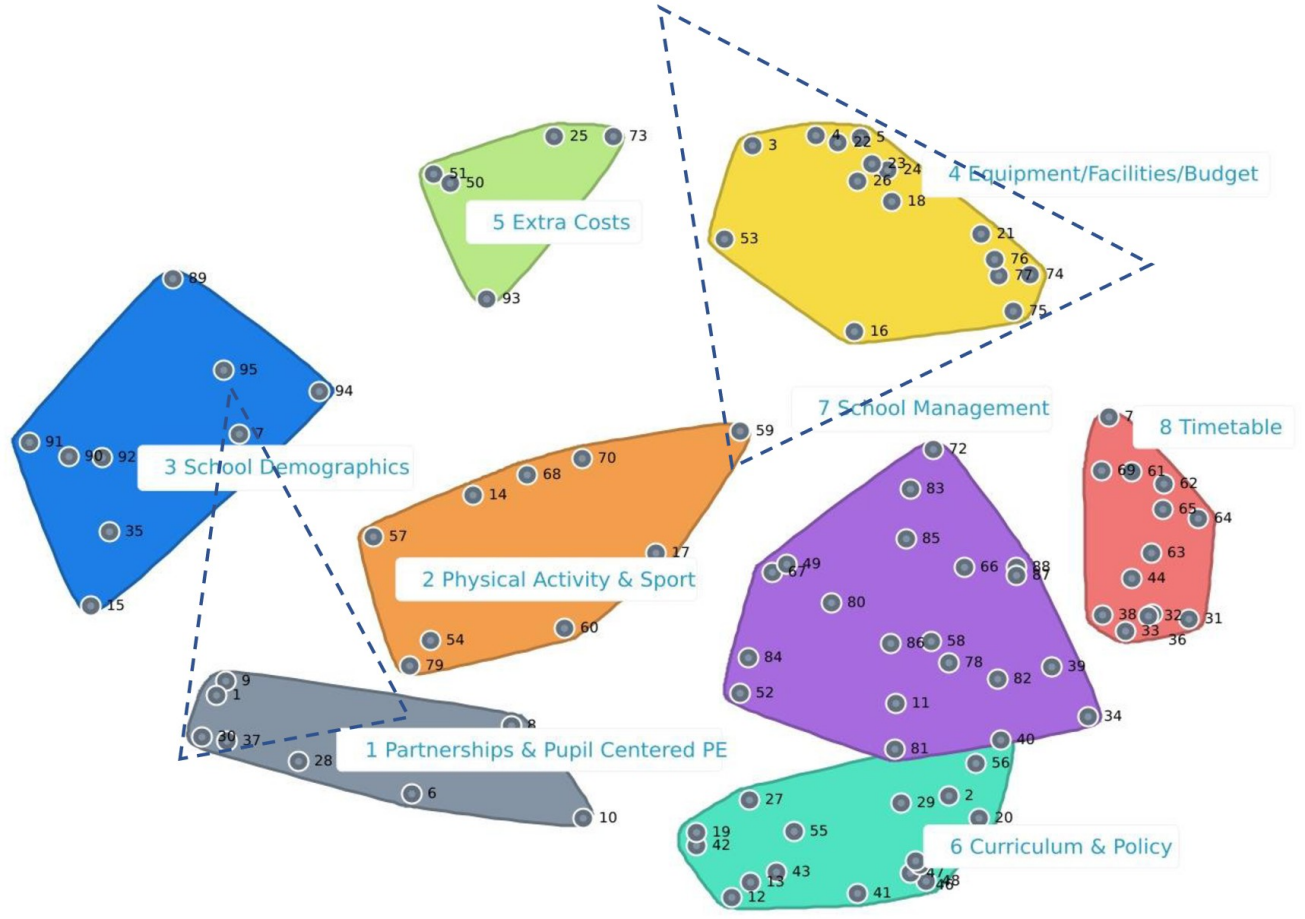
Cluster map.

Eleven iterations were required during the multi-dimensional scaling analysis of the similarity matrix. A stress value ranges between 0 and 1 with a lower score indicating a stronger association between the data and cluster map. The assigned stress value of the sorted data was 0.266, indicating good validity and aligns with thresholds underpinned by Rosas and Kane who found the optimum mean stress value to range between 0.17–0.34 [18]. The mean number of groups clustered together by the 114 experts was 8.5 and the range was 3–32. One participant left out one statement, a second participant left out two statements and a third participant left out 19 statements. Fig 2 presents the cluster map produced post analysis, including two statements that was reassigned to an adjacent cluster with a better conceptual fit. The expert stakeholders were consulted, and it was agreed that an eight-cluster map was the best fit and most parsimonious illumination of the content. Cluster labels were discussed and finalised and consensus on face validity was achieved. The eight cluster groups included 1) Partnerships and Pupil Centered Physical Education (8 statements), 2) Physical Activity and Sports (9 statements), 3) School Demographics (9 statements), 4) Equipment, Facilities, Budget (15 statements), 5) Extra Costs (5 statements), 6) Curriculum and Policy (17 statements), 7) School Management (20 statements) and 8) Timetable (13 statements). The distance between each factor statement point, indicates the degree of the relationship between the conceptual topics. For example, participants considered factor statement 5, “Access to effective audio-visual equipment”, and statement 22, “Access to small item equipment (e.g., balls rackets, nets)”, strongly connected as reflected by their proximity to one another on the cluster map. Participants considered factor statement 89, “The location of the school (rural, urban, suburban)”, and statement 41,” The curricular emphasis placed by the National Council for Curriculum and Assessment on physical activity promotion, recommendations and health”, less connected due to their respective location on the west and east side of the cluster map.

### Rating/Go Zone

Value patterns between importance and modifiability were illuminated by calculating average rating scores for both cluster and factor statements. The mean rating for importance on a 5-point Likert scale for all the participants was 3.92 ± 1.02 at the factor statement level. A total of 12 participants did not rate one statement and one participant did not rate 3, 9, 5, 4, 22 and 6 statements. At the cluster level, excluding the cluster on school demographics, mean importance ratings indicated that participants considered there to be strong levels of importance with an average rating above the middle point on a 5-point Likert scale (3.85) that ranged between 3.55 (Extra Costs) and 4.19 (School Timetable). At the factor statement level, a total of 88/95 factors were deemed important with an average rating above the middle point on a 5-point Likert scale that ranged between 3.11, “The Department of Education—Whole school Inspections” and 4.70, “The importance placed by the school on physical education, physical activity and sports participation”. The participants found factor statement 80 “The importance placed by the school on physical education, physical activity and sports participation” (4.70), factor statement 1, “Pupil participation in PE Classes—percentage of class that regularly participate” (4.63) and factor statement 53, “Sufficient access for pupils to physical education, physical activity and sports school facilities (Indoor, outdoor, offsite)” (4.63) to be the most important factors to impact physical education, physical activity and sports provision in schools. The cluster on school demographics (2.92) had a larger span ranging from 2.38, “The school type (secondary, community, comprehensive) to 4.06, “The percentage of pupil involvement in extra-curricular activities” and produced lower ratings on importance overall with 6 of the 9 factor statements scoring between 2–3 on the 5-point Likert scale.

The mean rating for modifiability for all the participants was 3.37 ± 1.22 at the factor statement level. A total of eight participants did not rate one statement, two participants did not rate two statements and one participant did not rate three statements. At the cluster level, excluding the cluster on school demographics and extra costs, participants considered there to be strong levels of modifiability at the cluster level with an average rating above the middle point of on a 5-point Likert scale (3.29) that ranged between 3.30 (Timetable) and 3.88 (Partnerships and Pupil Centered Physical Education). The mean modifiability rating for the cluster on school demographics (2.19) and extra costs (2.82) fell below the middle point on a 5-point Likert scale. At the factor statement level, a total of 74/95 factors were deemed modifiable with an average rating above 3 on a 5-point Likert scale for modifiability that ranged between 3.08, “Access to facilities outside school and 4.22. “The promotion of physical education, physical activity and sports related opportunities to all pupils. The participants found factor statement 52, “The promotion of physical education, physical activity and sports related opportunities to all pupils” (4.22), factor statement 82, “The prioritization of physical education, physical activity and sports within the school” (4.14) and factor statement 80, “The importance placed by the school on physical education, physical activity and sports participation” (4.13), to be the most modifiable factors to impact provision in schools. Once more, the cluster on school demographics (2.19), had a larger span ranging from 1.45, “The location of the school (rural, urban, suburban)” to 3.82, “The percentage of pupil involved in extra-curricular activities” and produced lower ratings overall with 7 of the 9 factor statements scoring between 1–2 on the 5-point Likert scale.

**Fig 3:**
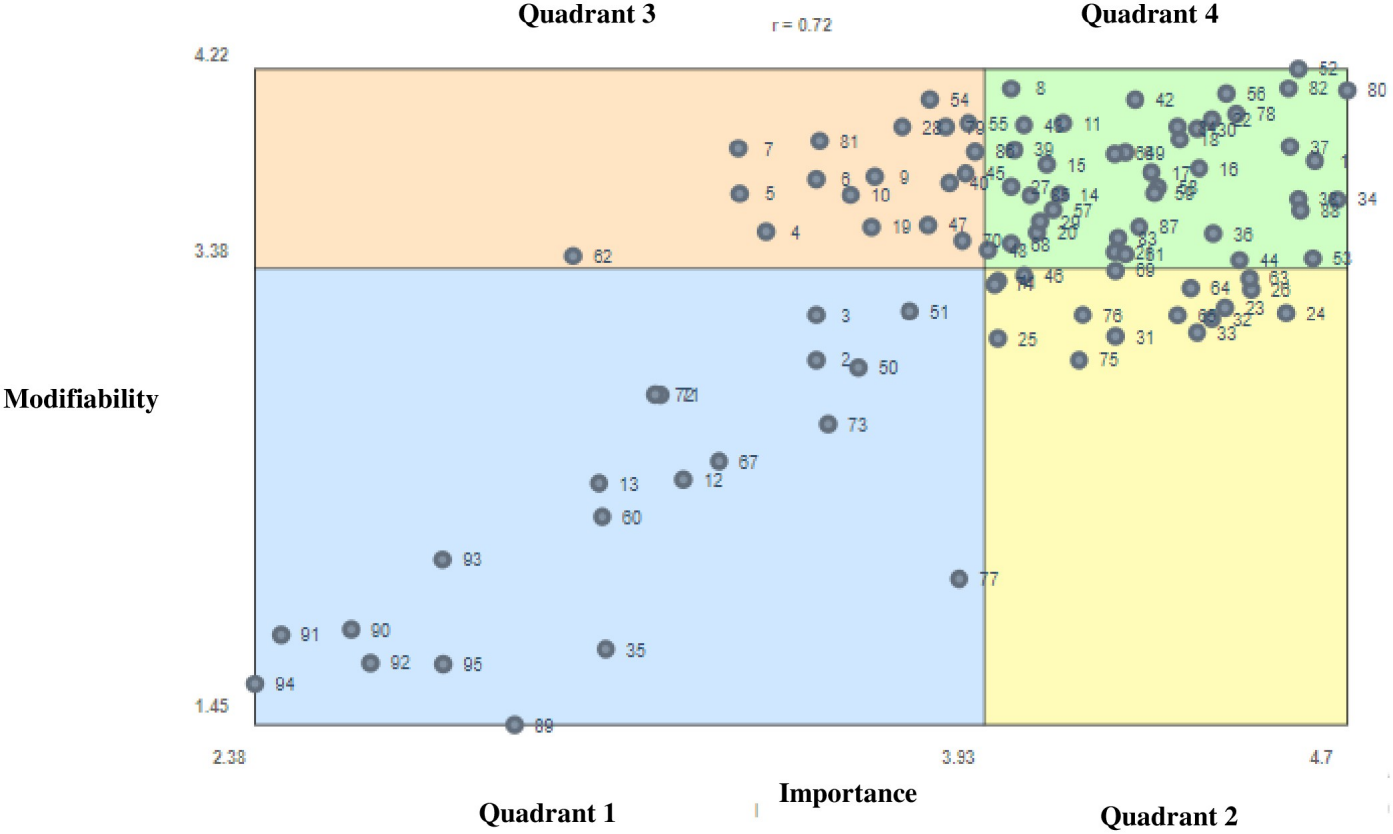
Go-Zone of factor statements that impact school physical education, physical activity and sports provision.

The Go Zone divides the factor statements into quadrants both below and above the mean for the importance and modifiability ratings and is illustrated in Fig 3. A moderate-strong positive correlation emerged between the importance and modifiability ratings at the factor statement level between all participants (r = 0.72). Of the 95 factor statements, 20 were located in quadrant 1 (below average importance and modifiability), 17 in quadrant 2 (above average importance, below average modifiability), 18 in quadrant 3 (below average importance and above average modifiability) and 40 in quadrant 4 (above average importance and modifiability), in relation to their impact on physical education, physical activity and sports provision. See Table 3 for mean importance and modifiability ratings and further interpretation of each Go-Zone quadrant. A separate Go-Zone for each individual cluster is provided in the manuscript S1 Fig.

**Table 3:** Clusters, mean importance and modifiability ratings and Go Zone quadrants for each factor statement.

Cluster Statement	Mean Importance	Mean Modifiability	Go Zone Quadrant
**Partnerships and Pupil Centered Physical Education**	4.03 ± 0.41	3.88 ± 0.14	
1. Pupil participation in Physical Education Classes—percentage of class that regularly participate.	4.63 ± 0.55	3.83 ± 0.90	4
37. The attitude of pupils to engaging in Physical Education, Physical Activity and Sports.	4.58 ± 0.63	3.89 ± 0.89	4
30. Pupil participation in Physical Education classes—percentage of class that regularly do not participate.	4.38 ± 0.83	3.97 ± 0.90	4
8. Communication and collaboration with parents/guardians.	3.99 ± 0.83	4.14 ± 0.84	4
9. The alignment, communication and collaboration with a Local Sports Partnership/Sports Ireland, Sports Clubs and National Governing Bodies of Sports.	3.70 ± 0.84	3.77 ± 0.90	3
28. Pupil input in to defining the nature and extent of school Physical Education provision.	3.76 ± 0.84	3.98 ± 0.90	3
6. Forming collaborative partnerships with higher education institutes in the context of provision (e.g., Teaching Council of Ireland, Physical Education Association of Ireland).	3.57 ± 1.01	3.76 ± 0.92	3
10. The Physical Education subject grade and report for pupils.	3.65 ± 0.97	3.69 ± 1.00	3
**Physical Activity and Sports**	3.93 ± 0.33	3.57 ± 0.48	
17. The provision of extra-curricular activities (e.g., number/range of extra-curricular activities weekly and time allocated).	4.28 ± 0.74	3.78 ± 0.97	4
59. The school personnel to support extra-curricular Physical Activity and Sports activities (e.g., exercise classes, coaching sports teams).*	4.29 ± 0.71	3.70 ± 0.91	4
14. The percentage of school personnel involved in extra-curricular activities.	4.09 ± 0.8	3.69 ± 1.01	4
57. The total number of school sports clubs available for pupil participation.	4.08 ± 0.94	3.63 ± 1.04	4
68. The number of voluntary school personnel who contribute to Physical Activity and Sports provision.	3.99 ± 0.88	3.48 ± 0.94	4
70. The number of school personnel who contribute to all aspects of the school on a voluntary basis.	3.88 ± 0.92	3.50 ± 1.01	3
54. The collaboration between school sports teams and the Physical Education department.	3.81 ± 1.06	4.09 ± 0.83	3
79. The emphasis on inter-school sports participation, competition and achievement.	3.85 ± 0.94	3.98 ± 0.82	3
60. The years of experience of Physical Education personnel teaching in current school.	3.12 ± 1.05	2.33 ± 1.17	1
**School Demographics**	2.92 ± 0.51	2.19 ± 0.90	
15. The percentage of pupil involved in extra-curricular activities.	4.06 ± 0.72	3.82 ± 0.88	4
7. Communication and collaboration with local leisure centers.*	3.41 ± 1.11	3.89 ± 0.96	3
35. Pupil age and year group.	3.13 ± 1.22	1.77 ± 1.18	1
95. The school size; small n = <300, medium n = 300–800, large = >800 (As per Department of Education guidelines).	2.78 ± 1.14	1.71 ± 0.94	1
90. The sex distribution within the school–more females.	2.58 ± 1.15	1.86 ± 1.13	1
89. The location of the school (rural, urban, suburban).	2.93 ± 1.2	1.45 ± 0.94	1
92. The school orientation -single sex/mixed school.	2.63 ± 1.25	1.71 ± 1.09	1
91. The sex distribution within the school–more males.	2.43 ± 1.11	1.83 ± 1.13	1
94. The school type (secondary, community, comprehensive).	2.38 ± 1.14	1.63 ± 1.06	1
**Equipment, Facilities, Budget**	4.14 ± 0.39	3.35 ± 0.45	
22. Access to small item equipment (e.g., balls, rackets, nets).	4.41 ± 0.6	4.01 ± 0.89	4
18. The regular maintenance of facilities and equipment.	4.34 ± 0.61	3.92 ± 1.00	4
16. The commitment of resources to the provision of extra-curricular activities as a priority (e.g., facilities and personnel).	4.39 ± 0.61	3.80 ± 0.89	4
53. Sufficient access for pupils to Physical Education, Physical Activity and Sports school facilities. (Indoor, outdoor, offsite).	4.63 ± 0.91	3.42 ± 1.13	4
21. The budget for renewal/purchase of small item equipment.	4.21 ± 0.7	3.45 ± 1.08	4
5. Access to effective audio-visual equipment.	3.41 ± 1.07	3.69 ± 0.95	3
4. Access to computers and recording equipment.	3.47 ± 1.16	3.53 ± 0.99	3
26. Consistent access to indoor and outdoor school facilities during timetabled hours for provision.	4.50 ± 0.59	3.29 ± 1.12	2
24. Fit for purpose indoor facilities (In compliance with The Department of Education Physical Education Hall and ancillary equipment list and specifications).	4.57 ± 0.57	3.19 ± 1.13	2
23. Fit for purpose outdoor facilities (In compliance with The Department of Education Physical Education Hall and ancillary equipment list and specifications).	4.44 ± 0.65	3.21 ± 1.15	2
76. The current budget available in the context of provision (e.g., equipment, field trips).	4.14 ± 0.66	3.18 ± 1.10	2
74. Additional sources of the budget attained by the school (non-Department of Education related) and the percentage given towards provision.	3.95 ± 0.81	3.31 ± 0.99	2
75. The Department of Education budget attained by school and percentage given toward provision.	4.13 ± 0.76	2.99 ± 1.18	2
3. Reliable broadband and internet access.	3.57 ± 1.17	3.18 ± 1.19	1
77. Past investments made in context of provision.	3.88 ± 0.86	2.07 ± 1.20	1
**Extra Costs**	3.55 ± 0.41	2.82 ± 0.37	
25. Access to facilities outside of school.	3.96 ± 0.94	3.08 ± 1.12	2
51. The costs incurred by pupils for their participation in curricular or extra-curricular activities.	3.77 ± 0.59	3.20 ± 1.04	1
50. The variation in costs incurred by pupils for involvement in curricular or extra-curricular activities (e.g., hockey players may need a hockey stick, gum shield and shin guards while basketball players may only need trainers).	3.66 ± 0.96	2.96 ± 1.10	1
73. The costs associated with the use of offsite provision related needs.	3.60 ± 0.90	2.72 ± 1.04	1
93. The school fees -fee paying/non-fee-paying school.	2.78 ± 1.13	2.15 ± 1.091	1
**Curriculum and Policy**	3.87 ± 0.31	3.51 ± 0.47	
56. Policies to ensure inclusion of pupils with disabilities. (PEN)	4.44 ± 0.79	4.12 ± 0.90	4
42. The appropriate emphasis of Physical Education learning outcomes related to participation, promotion and health.	4.25 ± 0.72	4.09 ± 0.77	4
43. The effective integration of relevant theoretical content with practical aspects of the Physical Education curriculum.	4.01 ± 0.81	3.99 ± 0.87	4
48. The adherence to the National Council for Curriculum and Assessment junior cycle Physical Education framework and guidelines.	3.94 ± 0.77	3.46 ± 1.20	4
29. The Leaving Certificate Physical Education subject status.	4.05 ± 0.95	3.58 ± 1.07	4
27. The curricular alignment between Physical Education, Physical Activity and Sports provision and activity in and out-of-school settings.	3.99 ± 0.82	3.73 ± 0.90	4
20. Health and safety policies in the context of provision.	4.04 ± 0.92	3.53 ± 1.13	4
55. Policies to ensure gender equity.	3.90 ± 0.70	3.99 ± 0.84	3
45. The adherence to the Wellbeing junior cycle framework and guidelines.	3.89 ± 1.06	3.78 ± 1.14	3
40. Policies in relation to supporting and providing Physical Education related field trips (e.g., adventure activities).	3.86 ± 0.88	3.74 ± 0.86	3
47. The adherence to the National Council for Curriculum and Assessment senior cycle Physical Education framework and guidelines.	3.81 ± 0.86	3.56 ± 1.11	3
19. The challenge of implementing health and safety policy in the context of provision.	3.69 ± 0.95	3.55 ± 1.00	3
46. The adherence to the National Council for Curriculum and Assessment Leaving Certificate Physical Education specification and guidelines.	4.01 ± 0.91	3.46 ± 1.19	2
41. The curricular emphasis placed by the National Council for Curriculum and Assessment on Physical Activity promotion, recommendations and health.	3.96 ± 0.88	3.33 ± 1.21	2
2. Compliance with the General Data Protection Regulations (GDPR).	3.57 ± 1.16	2.99 ± 1.43	1
13. The Department of Education—Whole school Inspections.	3.11 ± 1,13	2.47 ± 1.25	1
12. The Department of Education—Physical Education subject inspection.	3.29 ± 1.11	2.49 ± 1.23	1
**School Management**	4.18 ± 0.41	3.74 ± 0.41	
52. The promotion of Physical Education, Physical Activity and Sports related opportunities to all pupils.	4.60 ± 0.53	4.22 ± 0.87	4
80. The importance placed by the school on Physical Education, Physical Activity and Sports participation.	4.70 ± 0.50	4.13 ± 0.90	4
82. The prioritization of Physical Education, Physical Activity and Sports within the school.	4.58 ± 0.60	4.14 ± 0.87	4
78. A whole school approach that underpins provision of Physical Education, Physical Activity and Sports (e.g., active classroom breaks, active recess and active transport).	4.47 ± 0.64	4.03 ± 0.86	4
34. The implementation of the Physical Education curriculum by qualified Physical Education teaching personnel.	4.68 ± 0.55	3.67 ± 1.18	4
84. Leadership within the school Physical Education department.	4.34 ± 0.77	3.98 ± 0.96	4
88. Supportive school management.	4.60 ± 0.63	3.62 ± 1.05	4
49. The time allocated to after school/extra-curricular Physical Activity and Sports.	4.23 ± 0.88	3.87 ± 0.93	4
11. The schools self-evaluation on the extent and effectiveness of provision (e.g., implementation of school Physical Education curriculum and Physical Activity & Sports plan)	4.10 ± 0.78	3.99 ± 0.78	4
66. In-service training participation for school personnel.	4.21 ± 0.78	3.86 ± 0.89	4
58. The implementation of Physical Education, Physical Activity and Sports by school personnel with recognized qualifications/awards.	4.30 ±	3.72 ± 1.00	4
39. School policies in relation to supporting and providing sports related field trips (e.g., sports team travelling to competition).	3.99 ± 0.87	3.88 ± 0.84	4
87. Effective executive school management.	4.26 ± 0.89	3.55 ± 1.10	4
83. The internal school structures and support systems.	4.21 ± 0.76	3.51 ± 1.00	4
85. Collective school roles and responsibility to support provision.	4.03 ± 0.78	3.69 ± 0.93	4
81. The published school Physical Activity and Sports plan/policy.	3.58 ± 0.97	3.92 ± 0.96	3
86. Regular within school consultation regarding provision.	3.91 ± 0.82	3.87 ± 0.85	3
72. The total number of personnel employed in school structure (Teaching, admin, support staff).	3.23 ± 1.19	2.85 ± 1.13	1
67. The administrative challenge with the process of gaining Garda Vetting to gain support of voluntary personnel.	3.37 ± 1.02	2.56 ± 1.20	1
**Timetable**	4.19 ± 0.46	3.30 ± 0.21	
38. The prioritization and Implementation of formal timetabled Physical Education hours.	4.60 ± 0.58	3.67 ± 1.11	4
44. The capacity of school time allocated to Physical Education to effectively implement the national curricula.	4.47 ± 0.64	3.41 ± 1.17	4
61. The weekly workload of all Physical Education personnel (e.g., teaching, extra-curricular activities, other duties).	4.23 ± 0.75	3.44 ± 1.02	4
62. The formal timetabled hours of non- qualified Physical Education personnel.	3.06 ± 1.16	3.43 ± 1.15	3
36. The identified timetabled hours for Physical Activity and Sports provision.	4.42 ± 0.73	3.53 ± 1.09	2
63. The formal timetabled hours of qualified Physical Education personnel.	4.49 ± 0.62	3.34 ± 1.21	2
64. The number of school personnel employed with Physical Education teaching qualification who contribute to Physical Education provision on a full or part-time basis.	4.37 ± 0.74	3.30 ± 1.15	2
32. The weekly minutes timetabled for the senior cycle Physical Education framework.	4.41 ± 0.62	3.17 ± 1.19	2
69. The number of employed school personnel who contribute to the provision of Physical Education, Physical Activity and Sports.	4.21 ± 0.76	3.37 ± 1.10	2
65. The number of school personnel employed with a Physical Education teaching qualification.	4.34 ± 0.76	3.18 ± 1.21	2
33. The weekly minutes timetabled for the junior cycle Physical Education framework.	4.38 ± 0.72	3.11 ± 1.24	2
31. The weekly minutes timetabled for the Leaving Certificate Physical Education specification	4.21 ± 0.93	3.09 ± 1.21	2
71. The number of school personnel who work on a full or part-time basis.	3.24 ± 1.13	2.85 ± 1.15	1

Notes: Rating Scales: 0 = least important/modifiable, 5 = most important/modifiable. Go-Zone Quadrants: 1 = Bottom left; 2 = bottom right; 3 = top left; 4 = top right.

*Reassigned from “Physical Activity and Sports” cluster.

**Reassigned from “School Demographics” cluster.

Significant main effects among the clusters based on importance and modifiability were established (Tables 4 and 5). In 28 cases for importance, a total of 13 significant main effects between the clusters were illuminated. Differences between the cluster on school demographics and all other clusters accounted for over 50% of the total significant main effects. In 28 cases for modifiability, a total of 17 significant main effects between the clusters were illuminated. Differences between the cluster on school demographics and all other clusters accounted for 35% of the total significant main effects. Differences between the cluster on extra costs and all other clusters also accounted for 35% of the total significant effects. Tables 4 and 5 provide further details on the significant differences between clusters.

**Table 4:** Significant differences between clusters on importance.

	**Partnerships & Pupil Centered PE**	**Physical Activity & Sports**	**School Demographics**	**Equipment, Facilities & Budget**	**Extra Costs**	**Curriculiculum & Policy**	**School Management**	**Timetable**
**Partnerships & Pupil Centered PE**		0.594	<0.001^**+**^	0.558	0.064	0.335	0.404	0.428
**Physical Activity & Sports**	0.594		<0.001^**+**^	0.185	0.100	0.652	0.130	0.146
**School Demographics**	<0.001^**+**^	<0.001^**+**^		<0.001^**+**^	0.026*	<0.001^**+**^	<0.001^**+**^	<0.001^**+**^
**Equipment, Facilities & Budget**	0.558	0.185	<0.001^**+**^		0.011*	0.042*	0.762	0.754
**Extra Costs**	0.064	0.100	0.026*	0.011*		0.122	0.005**	0.011**
**Curriculiculum & Policy**	0.335	0.652	<0.001^**+**^	0.042*	0.122		0.016*	0.041*
**School Management**	0.404	0.130	<0.001^**+**^	0.762	0.005**	0.016*		0.954
**Timetable**	0.428	0.146	<0.001^**+**^	0.754	0.011*	0.041*	0.954	

Significance (p<0.05)* Significance (p<0.01)** Significance (p<0.001)^+^

**Table 5:** Significant differences between clusters on modifiability.

	**Partnerships & Pupil Centered PE**	**Physical Activity & Sports**	**School Demographics**	**Equipment, Facilities & Budget**	**Extra Costs**	**Curriculiculum & Policy**	**School Management**	**Timetable**
**Partnerships & Pupil Centered PE**		0.090	<0.001^**+**^	<0.001^**+**^	<0.001^**+**^	0.007**	0.201	<0.001^**+**^
**Physical Activity & Sports**	0.090		<0.001^**+**^	0.267	0.006**	0.757	0.382	0.118
**School Demographics**	<0.001^**+**^	<0.001^**+**^		<0.001^**+**^	0.087	<0.001^**+**^	<0.001^**+**^	<0.001^**+**^
**Equipment, Facilities & Budget**	<0.001^**+**^	0.267	<0.001^**+**^		0.017*	0.324	0.013*	0.685
**Extra Costs**	<0.001^**+**^	0.006**	0.087	0.017*		0.002**	<0.001^**+**^	0.015*
**Curriculiculum & Policy**	0.007**	0.757	<0.001^**+**^	0.324	0.002**		0.135	0.103
**School Management**	0.201	0.382	<0.001^**+**^	0.013*	<0.001^**+**^	0.135		<0.001^**+**^
**Timetable**	<0.001^**+**^	0.118	0.001**	0.685	0.015*	0.103	<0.001^**+**^	

Significance (p<0.05)* Significance (p<0.01)** Significance (p<0.001)^+^

## Discussion

The aim of this study was to develop a framework of distinct structures and associated factors, which constitutes and impacts on school physical education, physical activity and sports provision. Eight overarching structures which account for school physical education, physical activity and sports provision have been identified. Within each of these, overall factors of greatest importance and modifiability have been illuminated. Previous studies incorporated quantitative based, survey driven data collection methods or qualitative thematic procedures. This is the first study to utilize a mixed-method group concept mapping approach, to address these methodological constraints by both rating and sorting factors that impact school physical education, physical activity and sports provision, based on themes and inherent value. The combined responses of the diverse cohort of participants with multi-disciplinary backgrounds, were capitalized to provide the foundation for the development of a national provision evaluation index which can inform both school level and national policy and actions. The provision evaluation framework presents recommendations, not conclusions. In all, research focus on factors located in Go-Zone quadrant 4 (above average importance and above average modifiability) may result in a more targeted approach in identifying factors and themes that are both important and modifiable to the provision of school physical education, physical activity and sports.

Of the 8 clusters, a minimum of one factor statement from each cluster, with the exception of the cluster on ‘Extra Costs’, was located in Go-Zone quadrant 4 (Fig 3). This finding is consistent with the postulation that multi-level relationships between components of school physical education, physical activity and sports provision exist [30, 31] and support prior calls from the literature for multi-strategic approaches to ensure successful implementation to enhance overall impact [32]. The bulk of the evidence in the cluster on extra ‘Extra Costs’ and ‘School Demographics’ were located in Go-Zone quadrant 1 and indicated that factors statements such as fee paying/non-fee-paying schools and pupil age and year group had little impact on provision.

### School management

The cluster on school management was deemed to have the greatest impact on provision, including the largest number of statements (20 factors), with the highest mean ratings for importance and modifiability. It is noteworthy, that overall, a considerable bulk of the evidence with the highest mean importance and modifiability ratings, were plotted in close approximation to the cluster on school management, illuminating close conceptual relations with this cluster. A total of 79% of the factor statements were located in Go-Zone quadrant 4. This is consistent with a recent study that found, one of the biggest factors impacting provision, to be institutional, from the top down, with “support from management” and “access to professional development from school management”, cited as a significant contributor, and equally a barrier to provision if not implemented correctly [33 p9]. In addition, four of the top five rated factor statements overall resided within the cluster on school management. The International Society for Physical Activity and Health (ISPAH) identified a whole school approach as a key factor to impacting the capacity of school physical education, physical activity and sports provision [17]. This is consistent with factor statement 78, that recognized a whole school approach as one of the most significant factors to impact provision. Literature has found that viable strategies to endorse a whole school approach include implementation of active transport, active recess, classroom breaks, extra-curricular activities, physical education and school sports [34]. Interestingly, the remaining three highest rated factors overall were “The importance placed by the school on physical education, physical activity and sports” (factor 80), “The promotion of physical education, physical activity and sports related opportunities to all pupils” (factor 52) and “the prioritization of physical education, physical activity and sports within the school” (factor 82). This suggests that school ethos plays a vital role in the extent to which physical education, physical activity and sports are provided. These findings support existing evidence that illuminates the “ethos of physical activity for life within the school” as a key contributor to provision [33 p3, 35, 36]. Furthermore, evidence suggests that school ethos can be enhanced via the commitment of resources, expertise and availability of staff, the provision of extra-curricular physical activity and sports and availability of equipment, facilities and budget [36].

### Equipment, facilities and budget

The present study found the cluster on equipment, facilities and budget to have a considerable impact on provision, with 5 of the factor statements (33%) residing in Go-Zone quadrant 4. The key theme of these factor statements pertains towards “access” to facilities and equipment for both students and personnel. The combined view of the participants advocated for access to small item equipment and school facilities both during and outside of timetabled physical education class, e.g., recess time. This is consistent with a recent qualitative study that examined barriers to providing physical education and physical activity, identifying accessibility of facilities and equipment as the greatest and 3^rd^ greatest barrier to provision [33]. In order for schools to endorse access to facilities and equipment, the literature suggests that management support is vital in advocating for access during scheduled physical education time but also in endorsing a rotated timetable of supervision and instruction, outside of scheduled physical education time e.g., extra-curricular activities [37]. Furthermore, building collaborative partnerships with local facilities and other nearby schools, that are reasonably straightforward to access, may be a cost-effective method to facilitate access to facilities and equipment for physical education, physical activity and sports both in and outside of school [37]. It is noteworthy, that the cluster on equipment, facilities, and budget also found the regular maintenance of facilities to be a key impacting factor on provision. This is consistent with the worldwide survey of school physical education that found overwhelming evidence on challenges associated with low levels of maintenance and the provision of physical education, with two-thirds of countries citing “that physical education is challenged by the low or poor levels of maintenance of existing teaching facilities” and equipment [38 p22]. As a result of low-level maintenance, quality of facilities and equipment has been largely described as “inadequate” and not fit for purpose (cited as important factors to impact provision–factor 23 and 24). Comparisons between the updated worldwide survey 6 years later indicate that trends remain the same [13]. Utilizing the cluster on school management as the umbrella, protocols for regular maintenance of school facilities and equipment should be paramount in providing “conducive environments that translate into quality” provision [39 p5]. In addition to school management, the literature indicates that the school budget, although most often categorised in Go-Zone quadrant 2 (indicating above average importance and below average modifiability), is also a key determinant in ensuring accessibility and regular maintenance of facilities and equipment [40, 41]. Furthermore, although the participants deemed the school budget to be above average importance, their perception was that that there was little room to modify existing budgets given toward provision. This concept should be further considered when seeking to engage with teaching personnel regarding school physical education, physical activity and sports provision budgets.

### Partnerships and pupil centered physical education

Outside of the top 5 factor statements, “the attitude of pupils to engaging in physical education, physical activity and sports” was deemed the 6^th^ highest rated factor based on mean importance and modifiability. This illuminates the need to ensure that students maintain a positive disposition to engaging in physical education, physical activity and sports. It is well documented that student attitude toward physical education, physical activity and sports is strongly correlated with perceived competence and enjoyment of physical activity [42, 43]. These findings call to attention the crucial role of the teacher in fostering learned environments that promote realistic, attainable task competency measures in a pleasant and positive setting to effect student attitude and in turn impact overall provision of school physical education, physical activity and sports [44].

### Physical activity and sports / timetabling

The present study found the cluster on Physical activity and Sports to have a significant impact on provision, with 5 of the factor statements (56%) residing in Go-Zone quadrant 4. The key themes that underpinned the bulk of these factor statements pertained to engagement from the school personnel and the subsequent implementation of school physical activity and sports, with a primary focus on extra-curricular activities. These findings are consistent with the postulation that teacher support is integral to the provision of extra-curricular physical activities and sports [45]. A recent study also indicated a strong correlation between the provision of extra-curricular activities, teacher support and the development of a positive school culture, in which students are more likely to engage in physical activity [46]. Viable strategies to enhance the school culture “lies within the relationships and relational trust that exists” between teachers and students, therefore, teacher support to enhance this relationship and promote a positive physical activity culture within the school is paramount [47 p430]. In addition, the frequency of extracurricular physical activity and sports provision is also dependent on teacher support. However, it is noteworthy, that a lack of pay and teacher burnout have been cited as significant barriers to teacher support [48, 49]. Thus, it is suggested, that schools incentivise teacher support to overcome such barriers, as a key factor to impact provision and enhance the overall physical activity and sports culture within the school, in which students are more likely to participate. A timetable for rotation may be a suitable strategy to combat teacher burnout and ensure multi-disciplinary support from all staff members. It must be noted that factors pertaining toward timetabling and weekly workload within the cluster on “Timetable” agree with this concept and were also highlighted as important key factors to impact provision. Interestingly, the participants deemed the bulk of the factor statements on timetabling for personnel as high average importance but below average modifiability, indicating the perception of fixed hours for teachers, with little negotiation for adjustments. School management may consider actively engaging with teaching personnel to navigate best strategies that take in to account the teacher’s current timetable and overall contribution to school life when seeking to enhance provision of physical education, physical activity and sports.

### Curriculum and policy

A total of 6 factor statements (37.5%) in the cluster on Curriculum and Policy resided in Go-Zone quadrant 4. Based on mean importance and modifiability, “policies to ensure inclusion of pupils with disabilities” was deemed the 5^th^ highest rated statement overall. Further policy statements in G-Zone quadrant 4 included policies underpinning health and safety. The overall emphasis of policy for the effective provision of school physical education, physical activity and sports is consistent with SHAPE America, who endorse both policy and curriculum as key components of quality provision [50]. Interestingly, a recent systematic literature review examining the essential components of physical education provision also found policy to be a significant impactor [51]. However, literature illuminates a gap between policy adherence and practice, citing barriers to implementation such as a lack of support, goal priority (emphasis placed on one policy over another) and the teachers skillset to implement policy effectively [52]. Strategies to enhance policy implementation in schools may consider staff training to strengthen skills of implementation, school leadership endorsement and “systems to monitor implementation performance” [52 p7]. Interestingly, in service training, support systems and leadership in the cluster on “school management”, were all located in Go-Zone quadrant 4, substantiating the interrelatedness between the clusters. There was an assortment of factor statements that pertained to the curriculum element of this cluster in Go-Zone quadrant 4, in the context of physical education, ranging from subject status, adherence to the National Council for Curriculum and Assessment and curricular alignment. This is consistent with the postulation that “physical education curriculum can be effected by many factors, some of which can assist or hinder delivery” and should also be considered when seeking to enhance overall school physical education, physical activity and sports provision [33].

### Limitations and strengths

Although the current study adhered to the detailed guidance provided by Kane and Trochim [29], there are several limitations associated with the group concept mapping methodology that must be noted. First, given the multi-phase approach associated with this methodology, increased participant burden can lead to difficulty in recruitment and retention of participants. Second, selection bias in terms of participant recruitment is a distinct possibility, for example the undergraduate PE students were recruited from a single source. Third, in terms of the development of the factor statement list via the expert stakeholders, prior knowledge is a key consideration that may have influenced the final inclusion of factors upon consensus. Fourth, limitations pertaining to the generalizability of the results due to non-random selection should be considered.

However, this study also had a number of strengths. The present investigation is the first group concept mapping study to examine factors that impact school physical education, physical activity and sports provision and produce an evaluation framework. The mixed methodology extends the breadth and range of inquiry, with multi layers of detailed information via brainstorming, sorting, rating and analysis of the data. The group concept mapping methodology is replicable, includes evidence of validity and reliability and has the capacity to integrate the views of multi-disciplinary stakeholders into a composite conceptual framework. Finally, the prospects in utilizing the data acquired via the group concept mapping process to develop a measurement tool are substantial.

## Conclusion

The group concept mapping methodology has delivered additional understanding into the key factors and conceptual relationships that impact school physical education, physical activity and sports provision. The composite insights of the key stakeholders were consolidated into a framework that calls to attention the multitude of factors that impact provision. Eight overarching structures which account for school physical education, physical activity and sports provision have been identified. Within each of these, structures and overall factors of greatest importance and modifiability have been illuminated. The aforementioned findings, stemming from a rigorous methodology provide the foundation for the development of a national provision evaluation index which can inform school level and national policy and actions. It is recommended the current methodology is replicated in other nations to gain corresponding insights. Such research is essential to establish whether school physical education, physical activity and sports provision: 1) impacts key outcome measures such as physical activity behaviour, health and wellbeing of adolescents, 2) warrants sustained and/or greater financial investment, 3) requires modification of existing provision to potentiate positive impact and 4) provides an impetus for country comparison and benchmarking on key components of provision.

## Supporting information

S1 Table Factor statement list. (DOCX)

S1 Fig Go Zone quadrants for each individual cluster. (DOCX)
