# Supplementary material for: School-based physical education, physical activity and sports provision: A concept mapping framework for evaluation
Source: PLoS One. 2023 Jun 23;18(6):e0287505. doi: 10.1371/journal.pone.0287505 (PMC10289340; doi:10.1371/journal.pone.0287505)
Supplement: S1 Table — (DOCX) [file pone.0287505.s001.docx]

| Statement number |  |
| --- | --- |
| 1 | Pupil participation in Physical Education Classes - percentage of class that regularly participate. |
| 2 | Compliance with the General Data Protection Regulations (GDPR). |
| 3 | Reliable broadband and internet access. |
| 4 | Access to computers and recording equipment. |
| 5 | Access to effective audio-visual equipment. |
| 6 | Forming collaborative partnerships with higher education institutes in the context of provision (e.g. Teaching Council of Ireland, Physical Education Association of Ireland). |
| 7 | Communication and collaboration with local leisure centers. |
| 8 | Communication and collaboration with parents/guardians. |
| 9 | The alignment, communication and collaboration with a Local Sport Partnership/Sport Ireland, Sports Clubs and National Governing Bodies of Sport. |
| 10 | The Physical Education subject grade and report for pupils. |
| 11 | The schools self-evaluation on the extent and effectiveness of provision (e.g. implementation of school Physical Education curriculum and Physical Activity & Sport plan) |
| 12 | The Department of Education - Physical Education subject inspection. |
| 13 | The Department of Education - Whole school Inspections. |
| 14 | The percentage of school personnel involved in extra-curricular activities. |
| 15 | The percentage of pupil involved in extra-curricular activities. |
| 16 | The commitment of resources to the provision of extra-curricular activities as a priority. E.g. facilities and personnel. |
| 17 | The provision of extra-curricular activities (e.g. number/range of extra-curricular activities weekly and time allocated). |
| 18 | The regular maintenance of facilities and equipment. |
| 19 | The challenge of implementing health and safety policy in the context of provision. |
| 20 | Health and safety policies in the context of provision. |
| 21 | The budget for renewal/purchase of small item equipment. |
| 22 | Access to small item equipment (e.g. balls, rackets, nets). |
| 23 | Fit for purpose outdoor facilities (In compliance with The Department of Education Physical Education hall and ancillary equipment list and specifications). |
| 24 | Fit for purpose indoor facilities (In compliance with The Department of Education Physical Education hall and ancillary equipment list and specifications). |
| 25 | Access to facilities outside of school. |
| 26 | Consistent access to indoor and outdoor school facilities during timetabled hours for provision. |
| 27 | The curricular alignment between Physical Education, Physical Activity and Sport provision and activity in and out-of-school settings. |
| 28 | Pupil input in to defining the nature and extent of school Physical Education provision. |
| 29 | The Leaving Certificate Physical Education subject status. |
| 30 | Pupil participation in Physical Education classes - percentage of class that regularly do not participate. |
| 31 | The weekly minutes timetabled for the  Leaving Certificate Physical Education specification. |
| 32 | The weekly minutes timetabled for the senior cycle Physical Education framework. |
| 33 | The weekly minutes timetabled for the junior cycle Physical Education framework. |
| 34 | The implementation of the Physical Education curriculum by qualified Physical Education teaching personnel. |
| 35 | Pupil age and year group. |
| 36 | The identified timetabled hours for Physical Activity and Sport provision. |
| 37 | The attitude of pupils to engaging in Physical Education, Physical Activity and Sport. |
| 38 | The prioritization and Implementation of formal timetabled Physical Education hours. |
| 39 | School policies in relation to supporting and providing sport related field trips (e.g. sport team travelling to competition). |
| 40 | Policies in relation to supporting and providing Physical Education related field trips (e.g. adventure activities). |
| 41 | The curricular emphasis placed by the National Council for Curriculum and Assessment on Physical Activity promotion, recommendations and health. |
| 42 | The appropriate emphasis of Physical Education learning outcomes related to participation, promotion and health. |
| 43 | The effective integration of relevant theoretical content with practical aspects of the Physical Education curriculum. |
| 44 | The capacity of school time allocated to Physical Education to effectively implement the national curricula. |
| 45 | The adherence to the Wellbeing junior cycle framework and guidelines. |
| 46 | The adherence to the National Council for Curriculum and Assessment Leaving Certificate Physical Education specification and guidelines. |
| 47 | The adherence to the National Council for Curriculum and Assessment senior cycle Physical Education framework and guidelines. |
| 48 | The adherence to the National Council for Curriculum and Assessment junior cycle Physical Education framework and guidelines. |
| 49 | The time allocated to after school/extra-curricular Physical Activity and Sport. |
| 50 | The variation in costs incurred by pupils for involvement in curricular or extra-curricular activities (e.g. hockey players may need a hockey stick, gum shield and shin guards while basketball players may only need trainers). |
| 51 | The costs incurred by pupils for their participation in curricular or extra-curricular activities. |
| 52 | The promotion of Physical Education, Physical Activity and Sport related opportunities to all pupils. |
| 53 | Sufficient access for pupils to Physical Education, Physical Activity and Sport school facilities. (Indoor, outdoor, offsite). |
| 54 | The collaboration between school sports teams and the Physical Education department. |
| 55 | Policies to ensure gender equity. |
| 56 | Policies to ensure inclusion of pupils with disabilities. |
| 57 | The total number of school sports clubs available for pupil participation. |
| 58 | The implementation of Physical Education, Physical Activity and Sport by school personnel with recognized qualifications/awards. |
| 59 | The school personnel to support extra-curricular Physical Activity and Sport activities (e.g. exercise classes, coaching sport teams). |
| 60 | The years of experience of Physical Education personnel teaching in current school. |
| 61 | The weekly workload of all Physical Education personnel (e.g. teaching, extra-curricular activities, other duties). |
| 62 | The formal timetabled hours of non- qualified Physical Education personnel. |
| 63 | The formal timetabled hours of qualified Physical Education personnel. |
| 64 | The number of school personnel employed with Physical Education teaching qualification who contribute to Physical Education provision on a full or part-time basis. |
| 65 | The number of school personnel employed with a Physical Education teaching qualification. |
| 66 | In-service training participation for school personnel. |
| 67 | The administrative challenge with the process of gaining Garda Vetting to gain support of voluntary personnel. |
| 68 | The number of voluntary school personnel who contribute to Physical Activity and Sport provision. |
| 69 | The number of employed school personnel who contribute to the provision of Physical Education, Physical Activity and Sport. |
| 70 | The number of school personnel who contribute to all aspects of the school on a voluntary basis. |
| 71 | The number of school personnel who work on a full or part-time basis. |
| 72 | The total number of personnel employed in school structure (Teaching, admin, support staff). |
| 73 | The costs associated with the use of offsite provision related needs. |
| 74 | Additional sources of the budget attained by the school (non-Department of Education related) and the percentage given towards provision. |
| 75 | The Department of Education budget attained by school and percentage given toward provision. |
| 76 | The current budget available in the context of provision (e.g. equipment, field trips). |
| 77 | Past investments made in context of provision. |
| 78 | A whole school approach that underpins provision of Physical Education, Physical Activity and Sport (e.g. active classroom breaks, active recess and active transport). |
| 79 | The emphasis on inter-school sport participation, competition and achievement. |
| 80 | The importance placed by the school on Physical Education, Physical Activity and Sport participation. |
| 81 | The published school Physical Activity and Sport plan/policy. |
| 82 | The prioritization of Physical Education, Physical Activity and Sport within the school. |
| 83 | The internal school structures and support systems. |
| 84 | Leadership within the school Physical Education department. |
| 85 | Collective school roles and responsibility to support provision. |
| 86 | Regular within school consultation regarding provision. |
| 87 | Effective executive school management. |
| 88 | Supportive school management. |
| 89 | The location of the school (rural, urban, suburban). |
| 90 | The sex distribution within the school – more females. |
| 91 | The sex distribution within the school – more males. |
| 92 | The school orientation -single sex/mixed school. |
| 93 | The school fees -fee paying/non-fee paying school. |
| 94 | The school type (secondary, community, comprehensive). |
| 95 | The school size; small n=<300, medium n=300-800, large=>800 (As per Department of Education guidelines). |
