# Supplementary material for: School-based physical education, physical activity and sports provision: A concept mapping framework for evaluation
Source: PLoS One. 2023 Jun 23;18(6):e0287505. doi: 10.1371/journal.pone.0287505 (PMC10289340; doi:10.1371/journal.pone.0287505)

1) Partnerships and Pupil Centered PE (8 statements)


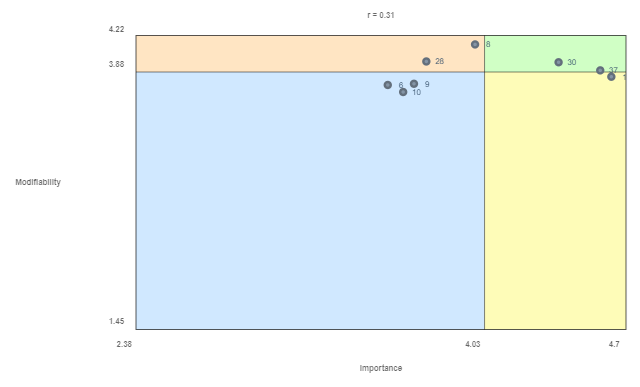


2) Physical Activity and Sport (9 statements)


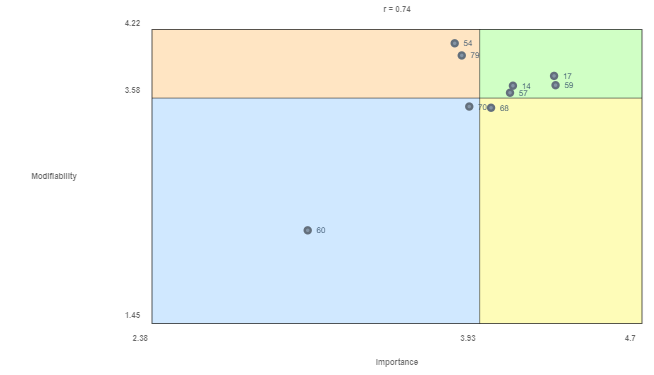


3) School Demographics (9 statements)


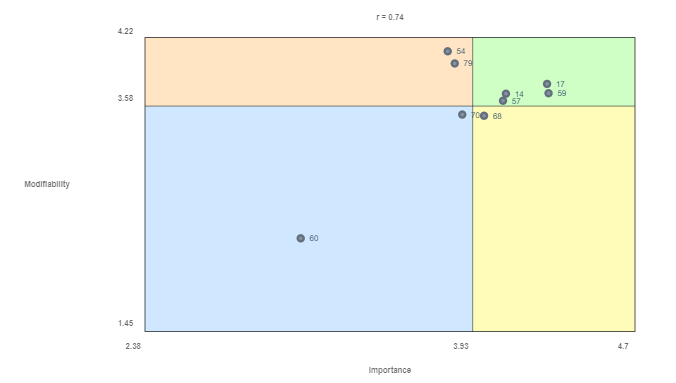


4) Equipment, Facilities, Budget (15 statements)


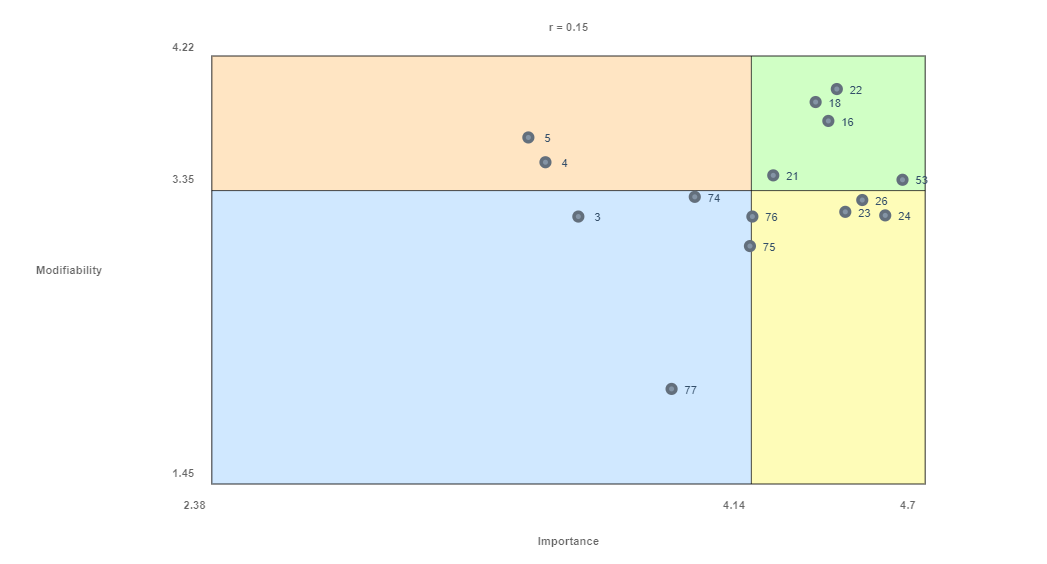


5) Extra Costs (5 statements)


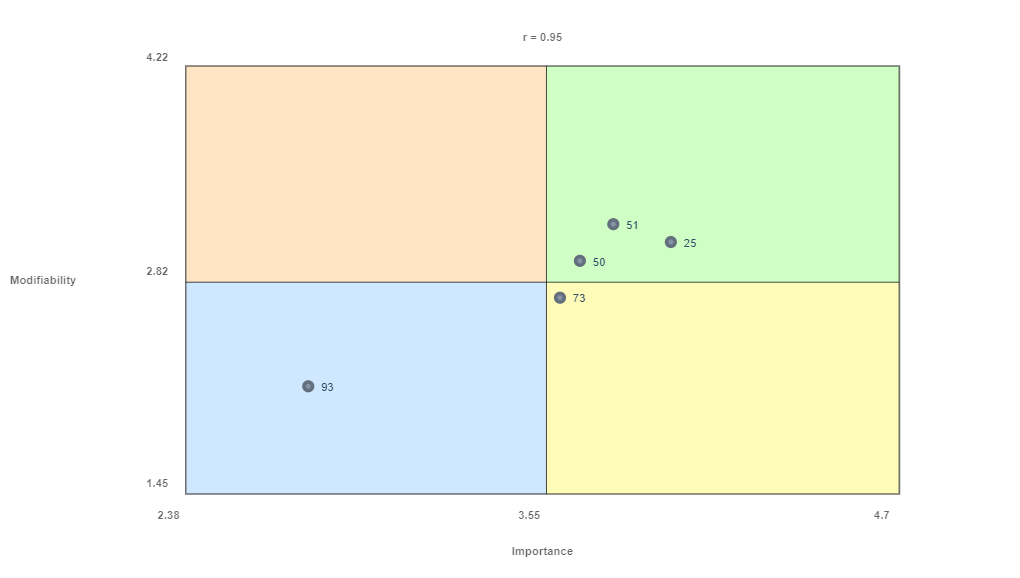


6) Curriculum and Policy (17 statements)


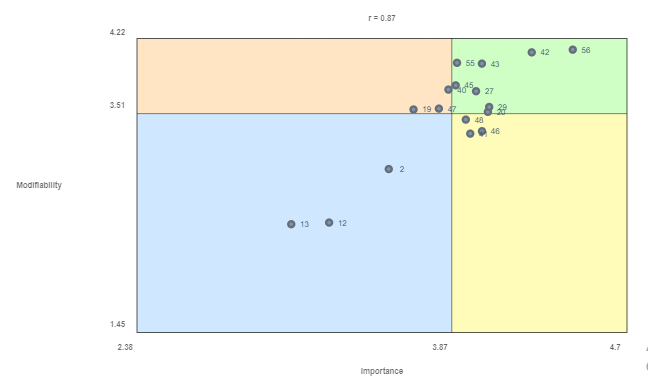


7) School Management (19 statements)


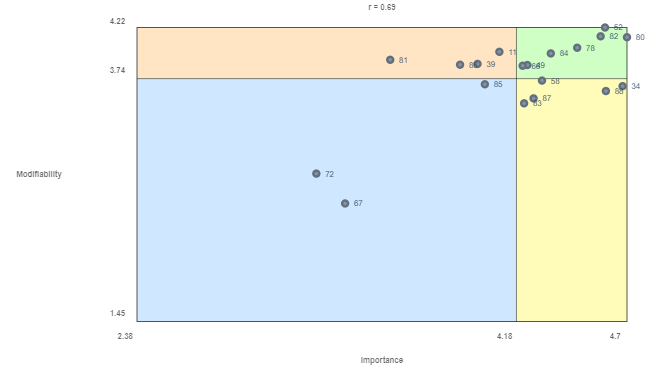


8) Timetable (13 statements).


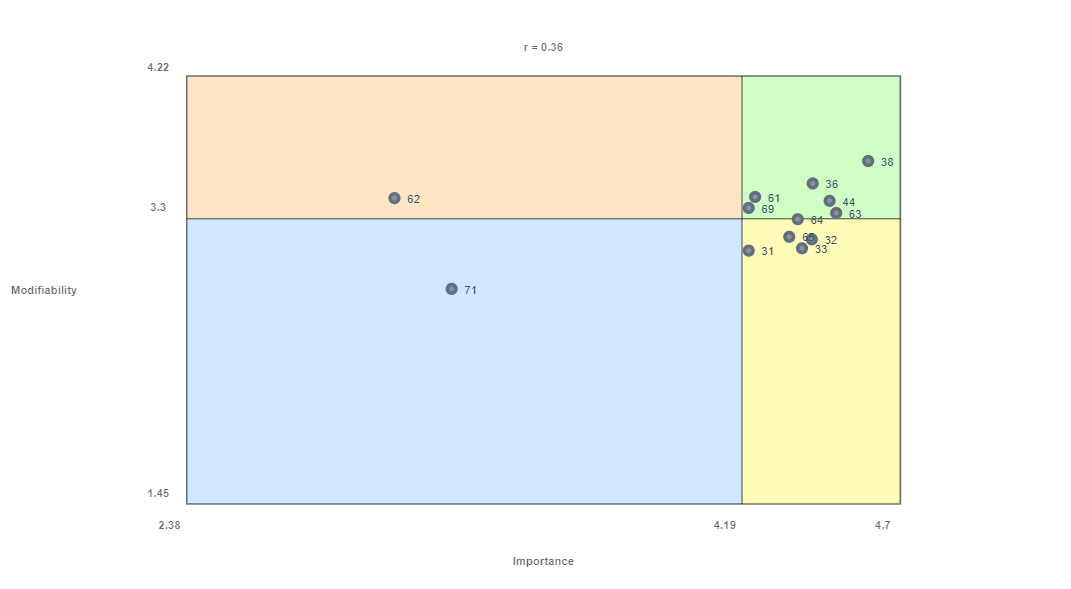

Supplement: S1 Fig — (DOCX) [file pone.0287505.s002.docx]
